# Complete Genome Sequence of Acinetobacter baumannii ATCC 19606^T^, a Model Strain of Pathogenic Bacteria Causing Nosocomial Infection

**DOI:** 10.1128/MRA.00289-20

**Published:** 2020-05-14

**Authors:** Taishi Tsubouchi, Masato Suzuki, Makoto Niki, Ken-Ichi Oinuma, Mamiko Niki, Hiroshi Kakeya, Yukihiro Kaneko

**Affiliations:** a Department of Bacteriology, Graduate School of Medicine, Osaka City University, Abeno, Osaka, Japan; b Research Center for Infectious Disease Sciences, Graduate School of Medicine, Osaka City University, Abeno, Osaka, Japan; c Antimicrobial Resistance Research Center, National Institute of Infectious Diseases, Aoba, Higashi-Murayama, Tokyo, Japan; d Department of Infection Control and Prevention, Osaka City University Hospital, Abeno, Osaka, Japan; Loyola University Chicago

## Abstract

Acinetobacter baumannii ATCC 19606^T^, which is often used in genetic studies as a routine model microorganism, belongs to sequence type 52 (ST52), showing beta-lactam resistance. We present the complete 3.996-Mbp genome sequence (1 chromosome plus 2 plasmids), generated by combining long-read (MinION) and short-read (MiniSeq) sequencing data.

## ANNOUNCEMENT

Acinetobacter baumannii ATCC 19606^T^, which was first reported in 1948 as an isolate from the urinary tract of a patient (1), was obtained from the American Type Culture Collection. A. baumannii is one of the leading causes of opportunistic nosocomial infections in immunocompromised hosts, and this human pathogen is responsible for a vast array of infections, of which ventilator-associated pneumonia and urinary tract and bloodstream infections are the most common. The mortality rate is serious and can reach up to 35% depending on the type of infection and the genetic background of the specific bacterial strain (2). A. baumannii is also known as one of the ESKAPE (*Enterococcus faecium*, *Staphylococcus aureus*, *Klebsiella pneumoniae*, *Acinetobacter baumannii*, *Pseudomonas aeruginosa*, and *Enterobacter*) pathogens and tends to acquire resistance to various kinds of antibiotics relatively easily; therefore, this species is an emerging threat for humans (3). Here, we announce the complete genome sequence of A. baumannii ATCC 19606^T^.

Strain ATCC 19606^T^, which had been stored at −80°C, was streaked onto a Mueller-Hinton II agar (BD) plate and incubated at 37°C for about 16 h. Genomic DNA extraction was performed using the Genomic-tip 20/G kit (Qiagen). Whole-genome sequencing was performed using the MinION system (Oxford Nanopore Technologies [ONT]) and the MiniSeq system (Illumina) with a high-output reagent kit (300 cycles). The library for MinION sequencing was prepared using a rapid barcoding kit (SQK-RBK004), and the library for Illumina sequencing was prepared using the Nextera XT DNA library prep kit. In this study, default parameters were used for all software except where otherwise noted. Base calling of ONT reads was performed using Guppy v3.2.1. The MinION reads were filtered using Filtlong v0.2.0 (https://github.com/rrwick/Filtlong; lengths of ≥1,000 bp and quality scores of ≥10) to keep only the top 90% of reads, with a target output of 500 Mbp (*N*_50_, 11,965 bp). These filtered reads were assembled *de novo* into three contigs using Canu v1.8 (4). The overlap region in the assembled contig was detected by a genome-scale sequence comparison using LAST (http://last.cbrc.jp) using the scaffold sequence for ATCC 19606 (GenBank accession number GCA_000737145) and was trimmed manually. Subsequently, long reads were mapped against the assembled genome with Racon v1.3.1.1 (5) twice to generate consensus sequences. These three contigs were combined with MiniSeq data (a total of 1,685,105 paired-end reads), which were quality filtered using Trimmomatic v0.30 (quality scores of ≥30) (6), to correct sequencing errors and to detect single nucleotide polymorphisms (SNPs) and indels using Pilon v1.20.1 (7) twice with binary alignment map (BAM) files generated from MinION and Illumina reads. Using Minimap 2 v2.17 (8), 99.7% of MiniSeq reads were mapped onto the final assembly, and the final genome coverage was 125×. The complete genome sequence was constructed as a total sequence of 3,995,851 bp, with a mean G+C content of 39.2%. A total of 3,884 coding DNA sequences (CDSs) and 6 sets of rRNA clusters were annotated using the DDBJ Fast Annotation and Submission Tool (DFAST) server (9). Using Pilon, the *dnaA* gene on the chromosome and *rep*-like gene on the plasmid were the first genes. The quality of the assembled genome sequence was evaluated using Benchmarking Universal Single-Copy Ortholog (BUSCO) v3 (10) with the *Gammaproteobacteria* odb9 data set. All 452 BUSCOs were located in the assembled genome. These six 16S rRNA genes shared 98.8% to 99.6% similarity with strain ATCC 19606^T^ (GenBank accession number Z93435). The genome sequence analyses were performed using ResFinder v3.2 (11) and MLST v2.0 (12) with default parameters. Strain ATCC 19606^T^ was classified by multilocus sequence typing as sequence type 52 (ST52), and the beta-lactam resistance genes *bla*_ADC-25_ and *bla*_OXA-98_ and the sulfonamide resistance gene *sul2* were detected.

Our intention in publishing the complete genome sequence of strain ATCC 19606^T^ is to underpin studies of antimicrobial-resistant A. baumannii strains found in hospitals across the globe.

### Data availability.

The complete genome sequence of A. baumannii strain ATCC 19606^T^ has been deposited in DDBJ/ENA/GenBank under accession numbers AP022836, AP022837, and AP022838. The raw sequence data are available in the Sequence Read Archive under accession numbers DRX202781 and DRX202782.

## References

[1] Hugh R, Reese R 1968 A comparison of 120 strains of Bacterium anitratum Schaub and Hauber with the type strain of this species. Int J Syst Evol Microbiol 18:207–203. doi:10.1099/00207713-18-3-207.

[2] Antunes LC, Visca P, Towner KJ 2014 *Acinetobacter baumannii*: evolution of a global pathogen. Pathog Dis 71:292–301. doi:10.1111/2049-632X.12125. 24376225

[3] Pendleton JN, Gorman SP, Gilmore BF 2013 Clinical relevance of the ESKAPE pathogens. Expert Rev Anti Infect Ther 11:297–308. doi:10.1586/eri.13.12. 23458769

[4] Koren S, Walenz BP, Berlin K, Miller JR, Bergman NH, Phillippy AM 2017 Canu: scalable and accurate long-read assembly via adaptive k-mer weighting and repeat separation. Genome Res 27:722–736. doi:10.1101/gr.215087.116. 28298431 PMC5411767

[5] Vaser R, Sovic I, Nagarajan N, Sikic M 2017 Fast and accurate de novo genome assembly from long uncorrected reads. Genome Res 27:737–746. doi:10.1101/gr.214270.116. 28100585 PMC5411768

[6] Bolger AM, Lohse M, Usadel B 2014 Trimmomatic: a flexible trimmer for Illumina sequence data. Bioinformatics 30:2114–2120. doi:10.1093/bioinformatics/btu170. 24695404 PMC4103590

[7] Walker BJ, Abeel T, Shea T, Priest M, Abouelliel A, Sakthikumar S, Cuomo CA, Zeng Q, Wortman J, Young SK, Earl AM 2014 Pilon: an integrated tool for comprehensive microbial variant detection and genome assembly improvement. PLoS One 9:e112963. doi:10.1371/journal.pone.0112963. 25409509 PMC4237348

[8] Li H 2018 Minimap2: pairwise alignment for nucleotide sequences. Bioinformatics 34:3094–3100. doi:10.1093/bioinformatics/bty191. 29750242 PMC6137996

[9] Tanizawa Y, Fujisawa T, Nakamura Y 2018 DFAST: a flexible prokaryotic genome annotation pipeline for faster genome publication. Bioinformatics 34:1037–1039. doi:10.1093/bioinformatics/btx713. 29106469 PMC5860143

[10] Simão FA, Waterhouse RM, Ioannidis P, Kriventseva EV, Zdobnov EM 2015 BUSCO: assessing genome assembly and annotation completeness with single-copy orthologs. Bioinformatics 31:3210–3212. doi:10.1093/bioinformatics/btv351. 26059717

[11] Zankari E, Hasman H, Cosentino S, Vestergaard M, Rasmussen S, Lund O, Aarestrup FM, Larsen MV 2012 Identification of acquired antimicrobial resistance genes. J Antimicrob Chemother 67:2640–2644. doi:10.1093/jac/dks261. 22782487 PMC3468078

[12] Larsen MV, Cosentino S, Rasmussen S, Friis C, Hasman H, Marvig RL, Jelsbak L, Sicheritz-Pontén T, Ussery DW, Aarestrup FM, Lund O 2012 Multilocus sequence typing of total genome sequenced bacteria. J Clin Microbiol 50:1355–1361. doi:10.1128/JCM.06094-11. 22238442 PMC3318499

